# Economic Impact of Dengue Illness in the Americas

**DOI:** 10.4269/ajtmh.2011.10-0503

**Published:** 2011-02-04

**Authors:** Donald S. Shepard, Laurent Coudeville, Yara A. Halasa, Betzana Zambrano, Gustavo H. Dayan

**Affiliations:** Brandeis University, Waltham, Massachusetts; Sanofi Pasteur, Lyon, France; Sanofi Pasteur, Swiftwater, Pennsylvania

## Abstract

The growing burden of dengue in endemic countries and outbreaks in previously unaffected countries stress the need to assess the economic impact of this disease. This paper synthesizes existing studies to calculate the economic burden of dengue illness in the Americas from a societal perspective. Major data sources include national case reporting data from 2000 to 2007, prospective cost of illness studies, and analyses quantifying underreporting in national routine surveillance systems. Dengue illness in the Americas was estimated to cost $2.1 billion per year on average (in 2010 US dollars), with a range of $1–4 billion in sensitivity analyses and substantial year to year variation. The results highlight the substantial economic burden from dengue in the Americas. The burden for dengue exceeds that from other viral illnesses, such as human papillomavirus (HPV) or rotavirus. Because this study does not include some components (e.g., vector control), it may still underestimate total economic consequences of dengue.

## Introduction

Over the past four decades, dengue disease has become recognized as the world's most important mosquito-borne viral disease, emerging in countries previously considered free of disease and reemerging in countries where the disease was once controlled.^1^,^2^ Inadequate mosquito control and increasing urbanization and air travel have placed an estimated 3 billion inhabitants of the world's tropical areas and roughly 120 million travelers at risk of dengue infection each year.^2^–^5^ Globally, the projected number of annual dengue infection cases is 50–100 million, with approximately 24,000 deaths, mainly in children, and an estimated annual burden of 750,000 disability-adjusted life years (DALYs).^3^,^5^–^10^ About 36 million symptomatic cases are estimated to occur annually.^11^

The case of the Americas is of special interest. From the 1950s to the 1970s, the Americas were a virtually dengue-free zone because of the eradication of *Aedes aegypti* (the principal vector of dengue in this continent) in a continent-wide vector control campaign. Combined with the campaign's interruption in the early 1970s, the acceleration of uncontrolled urbanization with its associated waste management problems in many Latin American cities contributed to the extensive distribution of *Ae. aegypti* and the return of dengue virus circulation.^1^,^7^,^12^–^15^ During the last two decades, all tropical areas of Central and South America, as well as most of the Caribbean, have experienced a sharp increase in the incidence of both dengue fever (DF) and dengue hemorrhagic fever (DHF): almost 3 million dengue cases were officially reported in the Americas for the period of 2001–2005 compared with 2.1 million cases for the period of 1995–1999.^14^,^16^–^18^

Dengue ranks fifth in the list of neglected tropical diseases in the Americas in terms of DALYs.^19^ However, with weak, passive surveillance systems, the true burden may be underestimated. Serological surveys suggest the occurrence of millions of dengue infections annually in the region.^9^,^14^,^20^–^23^ Additionally, epidemic dengue occurs cyclically every 3–5 years, with evidence suggesting an increase in the magnitude and severity of cases with each new epidemic.^2^,^9^,^24^,^25^

The few economic and social evaluations of dengue published to date provide empirical estimates for only one or a small number of countries, and comparisons are limited by important methodological differences.^4^,^9^,^26^–^35^ The cost of dengue has not been estimated at the scale of the American continents. Such estimates, which are available for several other vaccine-preventable diseases, are useful for developers of drugs, vaccines, insecticides, and novel insect control approaches as well as international donors and national governments to prioritize their efforts.^36^–^39^ To help address this need, this paper estimates of the economic costs and DALYs lost because of this disease in the Americas.

## Materials and Methods

Five major components are needed for estimating the economic and disease burden of a disease from a societal perspective: (1) the number of reported dengue cases, (2) the degree of underreporting, (3) the direct and indirect costs per case, (4) the DALY burden per case, and (5) the country's demographic information.

### Reported dengue cases.

The number of dengue cases was obtained from the Pan American Health Organization (PAHO), the regional body of the World Health Organization (WHO) responsible for the 35 nations and 9 territories of the Americas.^16^ Dengue is subject to important year to year variations in incidence. To stabilize the projections, estimations reported in this paper were based on the average of DF, DHF, and fatal cases reported over the period of 2000–2007 under the PAHO case definition.^18^

**Figure 1.**
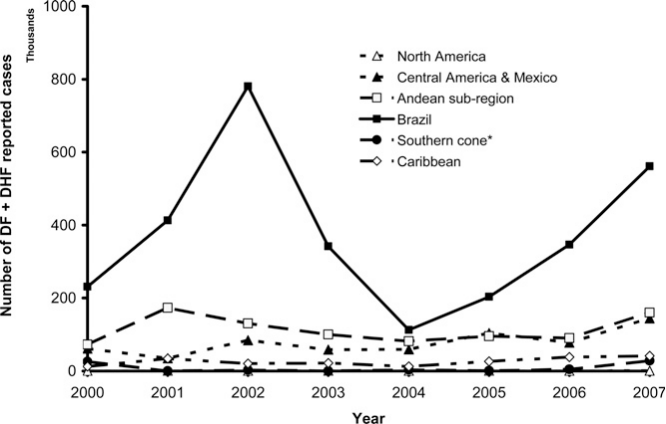
Number of dengue reported cases in the Americas from 2000 to 2007.

Countries were divided into six subregions: North America (United States of America and Canada), Central America and Mexico, the Andean region (Bolivia, Colombia, Ecuador, Peru, and Venezuela), Brazil, the Southern cone (Argentina, Chile, Paraguay, and Uruguay), and the Caribbean region. Reported dengue cases (DF and DHF) are presented in Figure 1.

The age distribution of cases, required for the estimation of DALYs and indirect cost of deaths, was available for Brazil, Mexico, Costa Rica, El Salvador, Honduras, Colombia, and Venezuela. For other countries in these three subregions, we applied the average country proportions from each subregion. For the three subregions with no country-specific information (Caribbean region, North America, and Southern cone), the average of all seven countries with available data was used.

### Dengue surveillance and underreporting.

Routine surveillance systems do not capture all dengue cases.^20^ To estimate the actual number of cases, reported cases need to be multiplied by a correction or expansion factor (EF), which represents the degree of underreporting.^40^ We identified five field studies in four American countries that provided sufficient information to estimate the underreporting rate (Table 1). Reporting of hospitalized cases (the severest cases) is relatively complete (range = 1.4–3.4).^41^–^43^ Reporting of milder, ambulatory cases is less complete. In a study in Nicaragua, combining all types of dengue cases, the EF ranged from 14 to 28.^44^ In addition to these five field studies, other authors provide estimates of underreporting. Luz and others^45^ recommended EFs for Brazil ranging from 1 to 10 for endemic years and from 0.3 to 10 for epidemic years using uniform distributions (central values = 5.5 and 5.15). Armien and others^27^ used an EF of 6 in estimating the costs of Panama's 2005 dengue epidemic. Meltzer and others^35^ used an EF of 10 for 0- to 15-year-old children and an EF of 27 for older children and adults in Puerto Rico.

We defined two EFs per country—one (EF1) for hospitalized and fatal cases and a second (EF2) for cases managed entirely in an ambulatory setting—and applied extensive sensitivity analyses to account for the uncertainty of these estimations. In countries with empirical estimates, EFs were defined in accordance with existing data. EF1 was set at 1.6 (range = 1.4–3.0) for Brazil, 2.3 (range = 1.4–3.3) for Colombia, Panama, and Nicaragua, and 2.4 (range = 2.3–3.4) for Puerto Rico. EF2 was set at 5 (range = 2.1–10) for Brazil, 9 (range = 4.5–18) for Colombia, 20.4 (range = 16–28) for Nicaragua, 6 (range = 3–12) for Panama, and 15 (range = 10–27) for Puerto Rico. In countries without empirical estimates, we used average expansion factors of 2.3 (range = 1.4–3.3) for EF1 and 15 (range = 9–28) for EF2.

### Dengue costs per case.

We conducted a systematic literature review for articles on dengue costs published through December 2009 using PubMed with keywords dengue and economics. From the 48 articles retrieved, 8 articles provided data covering nine locations in the Americas: Argentina,^30^ Brazil,^4^ Cuba,^9^,^28^,^31^ El Salvador,^4^ Guatemala,^4^ Nicaragua,^9^ Panama,^4^,^27^ Puerto Rico,^9^,^32^ and Venezuela.^4^,^26^ We also included a recent study about Puerto Rico presented at an international conference.^46^ Methodological variability limits the comparability of the data. Three studies were retrospective analyses of the costs of a large epidemic.^28^,^31^,^32^ Three other studies retrospectively estimated costs of infections from official records.^26^,^29^,^30^ One of these publications is a literature review,^9^ and two publications share cost data for Panama.^4^,^27^

**Figure 2.**
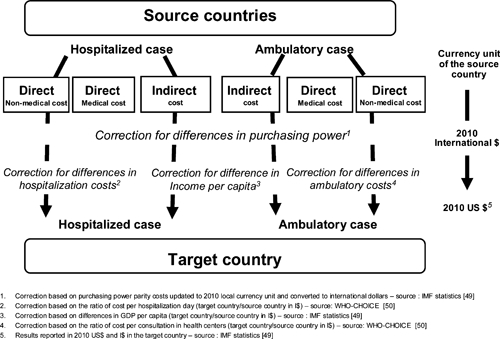
Method used for estimating cost of non-fatal dengue cases in all American countries.

The review found that available cost data were very limited. Also, the studies varied in approach and scope and were impacted by differences among the countries' health systems and economies.^47^,^48^ To develop consistent and comprehensive estimates, we needed to rely on studies that included both direct and indirect costs and funding from all sources (households, governments, and employers). Our analysis, therefore, focused on the results of two prospective cost studies for cost per dengue case: a large study with data corresponding to the year 2005 for Brazil, El Salvador, Guatemala, Panama, and Venezuela and a study performed in 2009 in Puerto Rico—conducted with similar methods as source countries.^4^,^46^ Both studies documented direct medical, direct non-medical, and indirect costs of dengue cases treated in hospital or ambulatory settings. For these six source countries, we updated the originally reported costs to 2010 US dollars (US$) using International Monetary Fund (IMF) statistics.^49^ For all other (target) countries, we used the average costs observed in source countries corrected for differences in purchasing power and cost structure (Figure 2).

We corrected for international differences in medical costs using the WHO-CHOosing Interventions that are Cost Effective (CHOICE) database.^50^ For hospital cases, the correction factor was based on the cost per hospitalization day, and for ambulatory cases, the correction factor was based on the cost per consultation in health centers (ratio of costs in target and source countries expressed in 2010 international dollars [I$]). Indirect costs were corrected for differences in income using the ratio of income per capita (expressed in I$) calculated using IMF statistics.^49^ Direct non-medical costs were corrected only for differences in purchasing power. Uncertainty in cost per dengue was accounted for by using the standard deviations observed in the multicountry cost study.^4^ We, therefore, assumed that cost per case followed a normal distribution, with coefficients of variation of 51% for ambulatory cases and 78% for hospitalized cases. All final results were expressed both in 2010 US$ and 2010 I$.

Consistent with reported observations, we considered that all DHF cases are treated in an inpatient hospital setting and that most (i.e., 90%; range = 80–100% in sensitivity analyses) DF cases are managed in an ambulatory setting.^4^ The economic cost of deaths was estimated using the human capital approach based on productivity losses.^51^ For each dengue death, the number of discounted life years lost was based on the age distribution of dengue deaths and remaining life expectancy at those ages in each country, which was obtained from WHO life tables.^52^ Productivity losses were calculated by weighting the discounted life years lost by the country-specific gross domestic product (GDP) per capita and using a 3% discount rate.^49^ Finally, the total cost of dengue illness in each country was obtained by combining data on unit cost per case with the corresponding number of cases corrected for underreporting.

### DALY calculations.

Disease burden was expressed in terms of standard DALYs and calculated in accordance with methods developed by WHO.^53^ The total number of years of life lost because of dengue-related death was estimated using the age distribution of dengue cases and age- and country-specific mortality rates.^52^ In accordance with previous studies, disability during non-fatal dengue cases was accounted for considering an average duration of 14 days for DHF (range = 10–18) and 4.5 days for DF (range = 2–7) and a disability weight of 0.81 for both DF and DHF.^35^,^45^,^54^

### Demographic data.

Results are presented both in absolute numbers and per unit of population (cases per 1,000 people and cost per capita). In all cases, we considered the entire population of the area under consideration for the results expressed per unit of population. Demographic data were drawn from international statistical databases of the US Census Bureau.^55^

### Sensitivity analyses.

The range of variation of the results of our analysis was assessed using probabilistic sensitivity analysis,^54^ including four categories of parameters: (1) the expansion factors (EF1 and EF2) with an associated distribution of triangular form based on country-specific values, (2) the proportion of DF cases treated in a hospital setting with a distribution of triangular form (mode = 10%; range = 0–20%), (3) the cost per dengue case using normal distribution with coefficients of variation of 51% for ambulatory cases and 78% for hospitalized cases, and (4) the duration of DF (range = 2–7 days) and DHF cases (range = 10–18 days) with an associated uniform distribution.^45^ For each sensitivity analysis, 5,000 Monte Carlo simulations were performed, with independent drawing for each parameter and each country. Results are presented as median values and 95% confidence intervals.

## Results

The estimated average annual number of dengue cases for the 2000–2007 period adjusted for underreporting is 5.6 million (Table 2). Following variations in reported cases, yearly projected numbers varied 3-fold, ranging from 2.6 million cases in 2004 to 7.8 million cases in 2007. Of these, there were an estimated 33,692 DHF cases (representing less than 1%) and an average of 453 deaths per year. Brazil contributed the highest number of cases (2.1 million; i.e., 39% of the total number of cases). Four of the six regions (Brazil, Andean region, Central America and Mexico, and the Caribbean) had similarly high incidence rates of about 1% per year.

The total cost per ambulatory case ranged from US$72 (Cuba) to US$2,300 (Bermuda), with a median value of US$472, whereas the total cost per hospitalized case ranged from US$306 in Nicaragua to US$17,803 in the United States (median value = US$1,227) (Table 3). The share of ambulatory case costs because of direct costs varies substantially between countries (e.g., 17% in Brazil and 95% in Puerto Rico). For most countries, the proportion of cost per case because of direct costs is higher for hospitalized cases than for ambulatory cases.

The aggregate annual total cost of dengue in the Americas for the period 2000–2007 was US$2.1 billion. When uncertainty on parameters values was incorporated, the estimate ranged from US$1–4 billion. Ambulatory cases accounted for 72.9% of the total costs overall, but the ambulatory share varied by 45.5% in North America (but over 60% in the five others subregions). About 60% of dengue costs in the Americas correspond to indirect costs. Most of these indirect costs are related to productivity losses induced by non-fatal dengue cases. Deaths accounted for only a small proportion (2.6%) of total costs of the disease.

**Figure 3.**
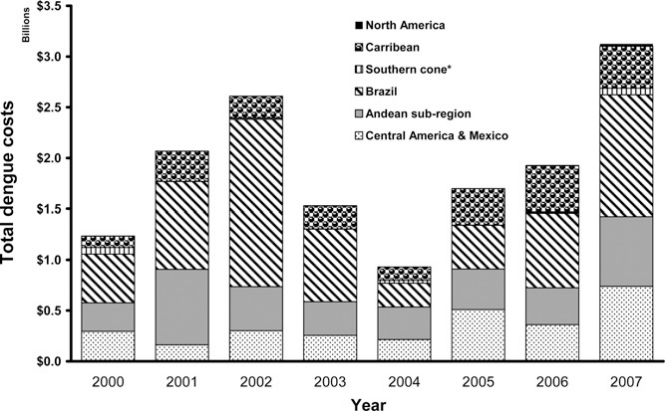
Annual economic burden in the Americas from 2000 to 2007 (in 2010 US$).

The geographic and temporal distribution of costs paralleled the distribution in numbers of reported cases. Geographically, Brazil alone accounted for 40.9% of the total cost of dengue, followed by the Andean region with 25.1%, Central America and Mexico with 17.7%, and the Caribbean with 15.0%. The Southern cone accounted for only 1.2% of the cost, and North America was less than 0.3%. When countries are considered individually, two additional countries stand out as major contributors to the dengue economic burden in the Americas: Venezuela (15%) and Mexico (7%). When costs were calculated separately by year, the annual cost of dengue ranged from $0.9 billion in a low incidence year (2004) to $3.1 billion in a high incidence year (2007) (Figure 3).

**Figure 4.**
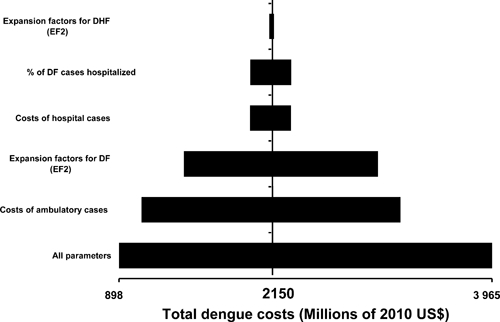
Variation of total dengue costs according to parameters included in the sensitivity analysis.

The two categories of parameters having the strongest impact on the variation of total dengue costs (Figure 4) in sensitivity analyses are (1) the cost per case of ambulatory cases, which generate a variation of total dengue costs from −55% to +55% and (2) the expansion factors for DF associated with a range of −33% to +40% of total dengue costs. This result is consistent with the fact that ambulatory cases are responsible for the major part of dengue economic burden.

The estimated cost per capita was highest in the four regions with the highest dengue annual incidence (about 1%), ranging from US$2.74 in Central America to US$8.29 in the Caribbean (Table 4).

The estimated number of DALYs lost each year for the entire region ranged from 45,080 to 115,874, with a median value of 72,277 (Table 4). Interestingly, the gap between Brazil and other areas is slightly lower when considering this criterion for assessing dengue burden, with 36% of the DALYs lost in Brazil, 28% in the Andean region, and 21% in Central America and Mexico. This result is mainly related to the number of deaths per dengue case (case fatality rate), which, in Brazil, is below the American average (5/100,000 versus 8/100,100). The number of DALYs per million inhabitants is 50–131 for the Americas as a whole or 90–233 if only the four regions at high dengue risk are considered.

The use of international dollars did not substantially modify the rank across regions (Table 5): Brazil remains the country where the cost of dengue is highest (42% of the total burden), and the Caribbean remains the area with the highest cost per capita (I$8.70). Aggregate costs in I$ are generally higher than their counterparts in US$, reflecting the differences in purchasing power between the United States and most of the American countries in which dengue is highly endemic. For example, aggregate costs for the Americas are I$3.2 billion (Table 5) compared with US$2.1 billion (Table 4).

## Discussion

Our analysis, combining available information on reported cases, levels of underreporting, and cost per case, shows that the annual economic burden of dengue in the Americas is substantial and subject to important year to year variation. About 60% of these costs are because of productivity losses (indirect costs), which affect households, employers, and governments. Brazil is the country with highest number of cases and highest cost, but the economic impact of dengue is substantial in many American countries, with a cost per capita greater than US$2 in four of the six American subregions considered (Andean region, Brazil, the Caribbean, and Central America and Mexico).

To our knowledge, this is the first assessment of the total cost of dengue illness for the Americas. Estimates have, however, been published for selected countries. Armien and others^27^ calculated that in 2005, a large epidemic year, dengue cost Panama about US$16 million. Our estimate for Panama is lower (about US$5 million), but our reference period differs (annual average over the 2000–2007 period), and unlike Armien and others,^27^ we did not include the cost of vector control efforts. Our estimate for Cuba is also lower than data reported by Guzman and others,^28^ but their report examined a year with a very large epidemic (1981). In contrast, annual costs reported by Suaya and others^4^ for five countries, Anez and others^26^ for Venezuela, and Torres and Castro^9^ for Nicaragua were lower than those in our analysis, but none of these authors corrected for underreporting. Comparing the results of different economic studies is a difficult exercise, requiring the understanding of both methodological and epidemiological differences. Our use of the same method in all American countries and the consideration of a reference period encompassing both high and low incidence years can, therefore, be viewed as a strength.

Our estimates of the annual number of DALYs related to dengue are consistent with previous reports, such as the WHO's assessment of the 2004 global burden of disease^53^ (73,000 DALYs for the Americas) and the estimate by Hotez and others^19^ (69,000 DALYs in 2006 for Latin America and the Caribbean). Comparing our results with those available for individual countries, our estimates are lower than the 658 DALYs per million inhabitants derived by Meltzer and others^35^ for Puerto Rico but higher than the 22 DALYs per million inhabitants reported by Luz and others^45^ for Brazil, although the wide range of possible values in this latter analysis included our point estimate.

Comparing the human and economic burden of dengue with that of other infectious diseases is informative. Dengue consequences, measured in DALYs, are substantial but lower than the results observed for some other diseases.^53^ Hotez and others^19^ ranked dengue fifth among neglected tropical diseases. Interestingly, Kim and others^36^ for rotavirus and Goldie and others^37^ for HPV also provided a point of comparison for countries in the Americas (Bolivia, Cuba, Guayana, Haiti, Honduras, and Nicaragua) eligible for Global Alliance for Vaccines and Immunization (GAVI) programs. The number of DALYs that can be prevented through vaccination against rotavirus (50,000) or HPV (31,000) is greater than the number of DALYs derived for dengue in our analysis for these GAVI countries (8,000), and the total cost of both rotavirus and HPV is estimated at I$10 million, which is more than 10-fold lower than the cost of dengue in our analysis (I$124 million). DALY calculations are, in fact, heavily driven by mortality, which fortunately, remains low for dengue (about 100 cases for the six GAVI countries). In absence of vaccination and with vector control as the only strategy to mitigate the spread of disease, dengue causes high incidence and morbidity, which is reflected in overall cost of the disease. Despite the lower number of DALYs, the potential economic attractiveness of vaccination might be as good, if not better, for dengue than for rotavirus or HPV.

The limitations of our analysis should not be overlooked. First, the expansion factors to correct for the underreporting of dengue cases are estimations based on limited datasets and expert opinion. Despite the unknowns, it is nevertheless critical to acknowledge underreporting in cost analyses to avoid systematically underestimating the disease burden. It is well-documented that routine surveillance systems, despite their critical role in the assessment and monitoring of disease burden, are not designed to comprehensively detect all cases.^56^ In such situations, extensive sensitivity analysis and conservative assumptions are advisable. The comparability of our estimations of dengue incidence in the four regions at high dengue risk—from 0.9% to 1.4%—with published data from prospective studies—0.4–1.8% in Nicaragua^56^ and 0.8% in Puerto Rico^57^—suggests that our assumed rates of underreporting were realistic.

The second limitation came from the absence of country-specific cost data for all American countries, which we managed by extrapolating data between countries in subregions and performing sensitivity analysis. Several factors might limit the validity of our extrapolation, including differences in patient and disease characteristics, differences in the national healthcare system, and differences in the overall level of costs.^47^,^48^ We nevertheless attempted to account for these factors. Specifically, the WHO-CHOICE database^58^ was used to consider differences in healthcare cost structure not related to differences in purchasing power. This goes beyond the usual approach based only on the correction of differences in purchasing power. The use of data collected with a consistent methodology is an added advantage in that process.

The third notable limitation is the exclusion of costs associated with dengue prevention (i.e., surveillance and vector control activities). Such costs have been found to add 43% to the cost of dengue in Panama,^27^ 49% in Puerto Rico,^46^ and 39% in Thailand.^59^ Data constraints similarly precluded the consideration of the impact of dengue outbreaks on tourism, potentially an important component.^60^ Finally, because the impact is often intangible, our analysis did not factor in the disruption to the rest of the health system caused by the seasonal clustering of dengue. For example, in Venezuela, a dengue epidemic country that publishes numbers of cases by week, the highest 13-week period (August 19 through November 17) contained more than three times as many cases (40%) as the lowest 13-week period (March 4 through June 9; only 12%).^59^,^61^,^62^ Therefore, our results may still underestimate the overall economic consequences of dengue, and the cost components excluded from the current analysis deserve study in the future. Finally, if the current trend of increasing dengue burden persists, total dengue costs are likely to grow over time, and the current estimate will need updating in a few years.

The major strength of our analysis is presenting estimates of dengue burden for all of the Americas combined as well as providing economic data not previously available for a large number of countries affected by dengue. The use of a consistent methodology for estimating costs in each country also facilitates the comparisons among countries of dengue burden. Our results are conservative, because some important dengue-related costs were not included. Nevertheless, these results enable a useful comparison of the economic consequences of dengue compared with other infectious diseases. The methodology developed here could be applied to dengue in other regions or in future updates. Such extensions would quantify the evolution of dengue in the Americas over time and the comparison of dengue with other conditions.

## Figures and Tables

**Table 1:** Sensitivity of dengue surveillance systems in American countries and corresponding expansion factors

Study	Country	Period	Type of dengue case	Method	Expansion factors
Duarte and Franca^42^	Brazil	1996–2002	Hospitalized dengue case	Sensitivity of the surveillance system using hospital records as the reference	Overall = 1.6 (1.4–1.8); DHF = 1.4 (1.3–1.5); DF = 2.1 (1.6–3.0)
Camacho and others^63^	Colombia	1995–1997	All types of dengue cases	Sensitivity of the surveillance system using emergency room medical records as the reference	Overall = 9
Standish and others^44^	Nicaragua	2004–2008	All types of dengue cases	Comparison of incidence obtained through active surveillance with reported incidence in the same area	Clinically diagnosed: 20.4 (2004–2008), 16 (2007–2008), 28 (2005–2006); lab-confirmed: 23.1 (2004–2008), 14 (2006–2007), 28 (2005–2006)
Dechant and Rigau-Perez^41^	Puerto Rico	1991–1995	Hospitalized dengue case	Capture–recapture method	1991–1995 = 2.4; 1991 = 2.3; 1993 = 3.4
Rigau-Perez^43^	Puerto Rico	1988–1997	Hospitalized dengue case	Sensitivity and specificity of the surveillance system compared with hospital records	Overall = 3

Figures in parentheses correspond to 95% confidence intervals assuming a binomial distribution for sensitivity analysis.

**Table 2:** Projected annual number of dengue cases in the Americas (2000–2007)*

Area	DF	DHF	Deaths	Cases per 1,000†
North America	1,692 (1,086–2,570)	0 (0–0)	0 (0–0)	0.005 (0.003–0.008)
Central America and Mexico	1,232,690 (1,002,139–1,490,177)	10,845 (8,623–13,123)	78 (66–89)	8.962 (7.28–10.83)
Andean subregion	1,621,678 (1,241,826–2,096,319)	20,173 (15,664–24,783)	100 (77–125)	13.715 (10.5–17.72)
Brazil	2,166,060 (1,109,386–3,464,314)	1,690 (1,295–2,422)	120 (92–172)	11.449 (5.87–18.31)
Southern cone‡	137,305 (92,782–201,479)	16 (11–21)	5 (3–7)	2.198 (1.49–3.23)
Caribbean	448,412 (381,315–524,059)	904 (779–1,036)	150 (122–179)	11.585 (9.85–13.54)
The Americas	5,607,836 (3,828,533–7,778,918)	33,628 (26,372–41,384)	453 (362–572)	6.355 (4.34–8.81)

Note that the intervals in parentheses show 95% confidence intervals.

* Annual average calculated over the 2000–2007 period.

† DF + DHF cases per 1,000 people.

‡ Countries other than Brazil.

**Table 3:** Estimated average cost per dengue case by country and setting in 2010 US$

Country	Ambulatory cases				Hospitalized cases			
	Direct medical	Direct non-medical	Indirect	Total cost	Direct medical	Direct non-medical	Indirect	Total cost
Andean subregion								
Bolivia	$95	$10	$51	$155	$143	$111	$137	$392
Colombia	$66	$11	$108	$185	$353	$128	$291	$772
Ecuador	$127	$11	$97	$235	$251	$125	$262	$638
Peru	$132	$11	$116	$259	$288	$123	$312	$723
Venezuela*	$118	$18	$194	$331	$864	$64	$310	$1,238
Central America								
Belize	$239	$13	$115	$367	$387	$144	$311	$842
Costa Rica	$279	$14	$174	$467	$503	$157	$469	$1,129
El Salvador*	$27	$2	$77	$107	$289	$170	$99	$559
Guatemala*	$24	$10	$78	$111	$304	$155	$72	$531
Guyana	$43	$9	$44	$95	$174	$98	$118	$389
Honduras	$52	$12	$60	$124	$179	$135	$161	$475
Mexico	$264	$13	$209	$486	$502	$144	$563	$1,209
Nicaragua	$108	$9	$27	$144	$133	$100	$74	$306
Panama*	$78	$25	$313	$416	$514	$419	$404	$1,336
Suriname	$92	$17	$172	$281	$435	$193	$463	$1,091
North America								
Canada	$360	$22	$963	$1,345	$2,036	$243	$2,599	$4,878
United States of America	$401	$23	$1,186	$1,610	$14,350	$254	$3,199	$17,803
Southern cone								
Argentina	$279	$12	$202	$493	$617	$138	$545	$1,300
Brazil*	$49	$18	$317	$383	$381	$47	$460	$889
Chile	$259	$12	$207	$478	$523	$136	$557	$1,216
Paraguay	$55	$11	$62	$128	$218	$127	$167	$512
Uruguay	$314	$15	$244	$574	$601	$170	$659	$1,430
The Caribbean								
Antigua and Barbuda	$274	$18	$390	$682	$762	$199	$1,051	$2,012
Bahamas	$336	$19	$553	$907	$1,198	$208	$1,491	$2,897
Barbados	$297	$18	$380	$695	$940	$199	$1,026	$2,164
Cuba	$26	$7	$39	$72	$134	$76	$106	$317
Dominica	$213	$12	$145	$370	$325	$134	$390	$849
Dominican Republic	$80	$15	$145	$239	$391	$162	$391	$944
French Guiana	$421	$27	$300	$749	$2,346	$305	$810	$3,460
Guadeloupe	$421	$27	$289	$737	$2,346	$305	$779	$3,430
Martinique	$421	$27	$520	$968	$2,346	$305	$1,402	$4,052
Grenada	$251	$13	$170	$434	$408	$151	$459	$1,018
Haiti	$125	$14	$21	$160	$98	$155	$57	$311
Jamaica	$71	$14	$127	$212	$289	$162	$342	$792
Aruba	$420	$26	$734	$1,180	$2,500	$291	$1,980	$4,771
Curaçao	$420	$26	$520	$966	$2,500	$291	$1,402	$4,193
Saint Kitts and Nevis	$352	$17	$271	$640	$663	$193	$730	$1,586
Saint Lucia	$237	$13	$162	$412	$361	$148	$437	$947
St. Vincent and Grenadines	$74	$13	$157	$244	$377	$147	$424	$948
Trinidad and Tobago	$505	$21	$530	$1,057	$1,183	$240	$1,430	$2,853
Anguilla	$334	$21	$1,052	$1,408	$1,929	$236	$2,839	$5,004
Bermuda	$334	$21	$1,944	$2,300	$1,929	$236	$5,246	$7,411
British Virgin Islands	$334	$21	$1,052	$1,408	$1,929	$236	$2,839	$5,004
Cayman Islands	$334	$21	$1,206	$1,561	$1,929	$236	$3,253	$5,418
Montserrat	$334	$21	$1,052	$1,408	$1,929	$236	$2,839	$5,004
Turks and Caicos Islands	$334	$21	$1,052	$1,408	$1,929	$236	$2,839	$5,004
American Virgin Islands	$118	$23	$429	$569	$4,207	$254	$1,157	$5,618
Puerto Rico*	$498	$27	$30	$555	$1,764	$124	$2,939	$4,827

* Countries with available information on cost per case.

**Table 4:** Annual costs and DALYs induced by dengue illness in the Americas in 2010 US$ (2000–2007)*

Area	Total costs (millions of US$)	Cost breakdown (%)			Cost per capita	Cost per case	DALYs
		Ambulatory cases	Hospital cases	Deaths			
North America	5.4 (1.8–10.7)	45.5 (0–83)	54.5 (17–100)	0.0 (0–0)	0.02 (0.01–0.03)	$3,154 (1,684–4,138)	18 (7–35)
Central America and Mexico	380.8 (212.1–596.7)	75.6 (56–86)	22.7 (12–41)	1.6 (1–3)	2.74 (1.53–4.3)	$307 (210–398)	15,424 (11,353–20,423)
Andean subregion	538.6 (271.3–877.5)	68.4 (40–84)	29.2 (14–57)	2.2 (1–4)	4.50 (2.27–7.33)	$326 (218–414)	20,223 (13,712–28,872)
Brazil	878.2 (178.8–1996.7)	78.3 (17–95)	19.5 (4–76)	2.0 (1–9)	4.64 (0.94–10.55)	$410 (164–577)	26,492 (11,722–52,947)
Southern cone†	25.4 (10.1–45.8)	74.4 (39–90)	24.5 (9–59)	1.1 (1–3)	0.41 (0.16–0.73)	$184 (109–227)	1,658 (856–3,009)
Caribbean	321.4 (224.5–438)	62.2 (47–74)	31.7 (20–46)	6.1 (4–9)	8.29 (5.79–11.29)	$713 (592–837)	8,957 (7,430–10,588)
The Americas	2,149.8 (898.4–3,965.4)	72.9 (34–88)	24.4 (10–61)	2.6 (1.5–6.8)	2.42 (1.01–4.47)	$382 (236–508)	72,772 (45,080–115,874)

Note that intervals in parentheses show 95% confidence intervals.

* Annual average calculated over the 2000–2007 period.

† Countries other than Brazil.

**Table 5:** Aggregate annual costs induced by dengue illness in 2010 US$ (2000–2007)*

Area	Total costs (millions of I$)	Cost breakdown (%)			Cost per capita	Cost per case
		Ambulatory cases	Hospital cases	Deaths		
North America	5.4 (1.8–10.7)	45.5 (0–83)	54.5 (17–100)	0.0 (0–0)	0.02 (0.01–0.03)	$3,154 (1,684–4,138)
Central America and Mexico	669.2 (386.4–1026)	75.5 (56–86)	22.7 (13–41)	1.7 (1–3)	4.82 (2.78–7.39)	$538 (384–683)
Andean subregion	803.1 (462.6–1254.7)	68.8 (46–82)	28.5 (15–50)	2.6 (2–5)	6.71 (3.86–10.48)	$489 (371–591)
Brazil	1,348.6 (269.9–3076.9)	78.3 (18–95)	19.4 (4–75)	2.1 (1–9)	7.12 (1.43–16.25)	$627 (248–878)
Southern cone†	49.3 (20.6–89)	74.1 (38–90)	24.9 (9–60)	1.1 (1–3)	0.79 (0.33–1.43)	$358 (222–441)
Caribbean	335.9 (239.4–449.4)	62.0 (47–73)	30.5 (20–44)	7.3 (5–11)	8.66 (6.17–11.59)	$745 (626–854)
The Americas	3,211.4 (1,380.8–5,906.9)	73.7 (36–88)	23.6 (10–59)	2.6 (1.5–6.7)	3.62 (1.56–6.65)	$571 (362–752)

Note that intervals in parentheses show 95% confidence intervals.

* Annual average calculated over the 2000–2007 period.

† Countries other than Brazil.
